# A Method of Reducing Salt Content in Fermented Soy Sauce Improves Its Flavor and Quality

**DOI:** 10.3390/foods13060971

**Published:** 2024-03-21

**Authors:** Shuang Zheng, Zhenbin Zhang, Xiuli Zhao, Wanning Li, Lihua Hou

**Affiliations:** 1 State Key Laboratory of Food Nutrition and Safety, Tianjin University of Science & Technology. No. 29, 13th Avenue, Tianjin Economic and Technological, Tianjin 300457, China; szheng13@163.com (S.Z.); zzbtjbc@163.com (Z.Z.); 15590869955@163.com (W.L.); 2 School of Nursing & School of Public Health, Yangzhou University, Yangzhou 225000, China; zhaoxiuli2010@126.com

**Keywords:** fermented foods, amino acid nitrogen, soy sauce, reduced salt, free amino acids, koji, flavor compounds

## Abstract

Most commercially available soy sauce is fermented by high-salt liquid-state (HS) fermentation, which has an excessive salt content and a long fermentation period. In this study, a new salt-reduced fermentation (SR) soy sauce technology involving multiple strains of bacteria was developed to reduce consumers’ salt intake. The SR soy sauce was found to have an amino acid nitrogen content of 8.40 g/L and over 80 kinds of flavor substances, which were significantly higher than those of low-salt solid-state fermented soy sauce and approximately equal to HS soy sauce. Compared with HS soy sauce, the salt content of the SR soy sauce was reduced by 59.2%, achieving the salt reduction goal. The proportion of umami amino acids in SR soy sauce reached 32.0% of the total level, enhancing SR soy sauce’s quality. Hence, the new fermentation process can decrease salt content and shorten fermentation time.

## 1. Introduction

As a customary Asian condiment, soy sauce, with its savory taste and distinctive aroma, is significant in daily culinary consumption [1]. Soy sauce is mainly fermented by two kinds of soy sauce fermentation methods, namely, the low-salt solid-state fermentation (LS) method and the high-salt liquid-state fermentation (HS) method. The LS method yields less flavorful soy sauce while possessing a shorter fermentation period and lower salt content [2]. The fermentation duration of the HS method is usually thrice that of the LS method, resulting in superior quality soy sauce, albeit with a higher salt content [3]. Therefore, commercially available premium soy sauces commonly use the HS method.

In general, the concentration of sodium chloride in soy sauce is around 18–20 *w*/*v*% [4]. The WHO recommends a daily intake of 5 g of NaCl [5]. However, excessive salt intake is the primary cause of high blood pressure, making it crucial to reduce daily salt consumption to maintain health. While salt plays a critical role in the fermentation process of soy sauce by influencing the growth of microorganisms, such as yeast and lactic acid bacteria (LAB), as well as the flavor and texture of the final product [6], it is not a simple matter to reduce the its concentration during fermentation [7]. 

Reducing salt in soy sauce can be achieved by three methods: postfermentation desalting, replacing sodium chloride with other chloride salts, or decreasing the salt content during fermentation [8,9]. Postfermentation desalination generally uses techniques such as nano-filtration, ion exchange, reverse osmosis, and extraction, but they are challenging to apply in factories due to the high operating costs [10]. Soy sauce produced using potassium chloride and calcium chloride has a bitter or unpleasant taste, and a study has shown that the bitterness becomes noticeable when potassium chloride is added at a concentration of more than 10.0% [11]. The direct reduction in salt content during fermentation can negatively affect a soy sauce’s fermentation and pose food safety issues [12], mainly resulting in reduced quality, poor aroma, and the proliferation of spoilage microorganisms in the soy sauce produced using a salt reduction technique, which is related to ecological disorders in the mash during fermentation. Therefore, there is an urgent need to study a salt-reduced fermentation (SR) soy sauce that is high quality and low cost. 

To enhance the flavor of soy sauce, LAB and yeast are added at different stages of the fermentation process. LAB is typically added during the early stages of soy sauce fermentation, helping to stabilize the pH and total acid values of the system, thereby restricting the growth of spoilage bacteria [13] and promoting the formation of soy sauce flavor and color during the later stages of fermentation by producing various organic acids [2]. At present, the fermentation of soy sauce mainly involves adding *Tetragenococcus halophilus* (*T. halophilus*), which can withstand high temperatures during soy sauce fermentation and enhance the aroma of soy sauce [14]. Several studies have reported that adding yeast during fermentation gives soy sauce a distinct aroma. Yeast strains, including *Zygosaccharomyces rouxii* (*Z. rouxii*), *Candida versatilis* (*C. versatilis*), and *Torulopsis halophilus* (*T. halophilus*), are commonly added to soy sauce during fermentation [15,16]. After approximately five months of fermentation, these microorganisms produce a significant number of flavor compounds, including alcohols, acids, esters, phenols, and other substances that impart soy sauce with a unique flavor [17].

The problems of hyperglycemia, hypertension, and hyperlipidemia have always plagued people. A diet high in salt is the main cause of high blood pressure, so reducing daily salt intake has become the primary task for people to ensure their health. Therefore, it is urgent to study an SR soy sauce that has good quality and low cost. Here, a new fermentation technology for SR soy sauce was studied, combining the LS method and HS method. This soy sauce can not only meet health requirements but also reduce the production costs for soy sauce enterprises.

This study investigated a new SR method for soy sauce, combining the LS and HS methods. The optimal combination and ratio of the raw materials were determined by the koji enzyme activity. Then, an ultrasonic treatment was applied to the soy sauce mash during the initial fermentation stage to expedite the breakdown of the raw materials and suppress the growth of extraneous bacteria. Subsequently, LAB and different yeast strains were added at different fermentation time points and fermentation temperatures. By evaluating a series of indexes, including flavor substance, free amino acids (FAAs) contents, organic acid content, and salt content, the quality of the SR soy sauce produced under the optimal fermentation conditions was compared to that of the LS- and HS-fermented soy sauce. 

## 2. Materials and Methods

### 2.1. Koji Production Process

The preparation process for *Aspergillus oryzae* (*A. oryzae*) seed koji referred to the method of Ding (2019) [18], and the preparation process for koji referred to the method of Zhang (2016) [19]. The koji stage was set up by mixing the ingredients at specific ratios (5:5, 6:4, and 7:3) and sterilizing them at 115 °C for 20 min for a soybean with fried wheat group and a soybean meal with fried wheat group. After sterilization, the temperature was reduced to 40 °C, and the koji was inoculated with 0.3% (*w*/*w*) *A. oryzae* 3.042. The koji was harvested after two days of incubation at 30–32 °C.

### 2.2. Soy Sauce Fermentation

After collecting the mash, a glass rod was used to stir the sauce mash to fully mix the brine with the mash. Then, the tank was sealed with plastic film and kraft paper and stored at a specific temperature for fermentation. To determine the optimal fermentation conditions, the effects of salinity (9%, 10%, and 11%), ultrasonic time (5 min, 7.5 min, 10 min, and 12.5 min) during the first three days, inoculation quantity (1.5 × 10^6^ CFU/g, 2.0 × 10^6^ CFU/g, and 2.5 × 10^6^ CFU/g) of the mixed *Z. rouxii* and *C. versatilis*, and temperature variation range (30~39 °C, 0~40°C, and 30~41 °C) on the amino acid nitrogen (AAN) content were studied. Regarding the above single-factor test results, three levels of response surfaces were tested for these four factors using Design-Expert 8.0 software, and the AAN content was used as the response value to determine the optimal fermentation process for the new SR soy sauce.

### 2.3. Analysis of Essential Indices

The AAN was measured according to Liu et al. [20]. A total of 20 mL of soy sauce diluent was mixed with 60 mL of pure water, and the mixed sample was titrated with 0.05 mol/L NaOH solution to pH 8.2. Then, 10 mL of formaldehyde solution was added. The mixture was then titrated with a 0.05 mol/L NaOH solution to a pH of 9.2. The content of amino acid nitrogen was calculated according to the volume of NaOH used. Total nitrogen (TN) was measured according to Zhang et al. [21]. Approximately, 2 mL soy sauce was added to the digestion tube and mixed with 0.2 g CuSO_4_, 3 g K_2_SO_4_, and 10 mL H_2_SO_4_ and heated to 450 °C for 4 h. The total nitrogen content was measured by an automatic Kjelter nitrogen detector (RK-9870, RayKol Co., Ltd., Xiamen, Fujian, China). The reducing sugar (RS) was determined by the 3,5-dinitrosalicylic acid (DNS) method [22]. A total of 1 mL of 20-fold diluted soy sauce dilution was mixed with 1 mL of DNS reagent in a test tube and kept warm in a water bath at 100 °C for 5 min. Later, the test tube was cooled to 25 °C, 5 mL of deionized water was added, and the absorbance was measured at 540 nm. The reducing sugar content was calculated according to the standard curve. The method for the determination of the salt content was slightly modified according to the method of Han et al. [23]. A total of 1 mL of diluted soy sauce was mixed with 99 mL of deionized water and 1 mL of 50 g/L K_2_CrO_4_. The mixture was titrated with 0.1 mol/L AgNO_3_ until the color of the mixture turned orange–red. The salt content was calculated on the basis of the volume of silver nitrate used.

### 2.4. Determination of Organic Acids Using High-Performance Liquid Chromatography (HPLC)

An HPLC method (LC-20A, Japan) was used to determine the contents of nine organic acids in soy sauce samples. A total of 10 mL of soy sauce sample was weighted, transferred to a 100 mL volumetric flask with deionized water, and the volume was adjusted to scale. After centrifugation of the diluted solution (8000 r/min, 20 min), the supernatant was passed through a 0.22μm microporous cellulose filter membrane and injected directly into the sample. The sample was separated on a HydrospHere C18 (250 mm × 4.6 mm I.D.) column at a flow rate of 0.50 mL/min using 0.03 mol/L of pH 3.0 KH_2_PO_4_ as the mobile phase. The UV detection wavelength was set to 215 nm. Nine organic acids were quantified using the extra peak area standard method.

### 2.5. Formation Rate of Amino Acid Nitrogen

The formula for calculating the formation rate of ANN is shown as follows:

(1) $$The\;formation\;rate\;of\;ANN=\frac{ANN}{TN}\cdot 100\%$$

where ANN is the amino acid nitrogen content, and TN is the total nitrogen content.

### 2.6. Analysis of Volatile Flavor Compounds Using Solid-Phase Microextraction/Gas Chromatography-Mass Spectrometry (SPME/GC-MS)

A sample of 5 mL of soy sauce was placed in a 20 mL headspace vial sealed using an aluminum cap. The sample was equilibrated for 20 min at 50 °C and extracted continuously for 30 min at 50 °C in a water bath using solid-phase microextraction fiber (50/30 mDVB/CARPDMS). The extraction tip was inserted into the inlet of the GC-MS (GCMS-QP2010 Ultra, Japan), and the fiber tip was pushed out and desorbed at 250 °C for 15 min. The column model used was VF-5MS, 30 m × 0.25 mm × 0.25 μm. GC conditions were as follows: helium carrier gas (99.999% purity), flow rate of 1 mL/min, shunt ratio of 5, and inlet pore temperature of 250 °C. Programmed temperature increase: the starting temperature was 40 °C, which was maintained for 10 min, increasing to 100 °C at 3 °C/min, then to 180 °C at 4 °C/min, and, finally, to 220 °C at 6 °C/min. MS conditions: the interface temperature was 220 °C, the temperature of the ion source was 230 °C, the solvent delay time was 1.5 min, the electron energy was 70 eV, and the mass range of the scan was 33~450 amu.

The data were collected with reference to the NIST 17 library and characterized by combining the retention time, mass spectrum, and retention index of the chemical components. The quantification was performed by the area normalization method with reference to the method of Li et al. [24].

### 2.7. Sensory Evaluation

A sensory evaluation was performed according to Steinhaus and Schieberle’s method [25], and the sensory characteristics of the soy sauces were compared. Ten students without visual, taste, and olfactory impairments (5 females and 5 males, aged 24–27 years) were invited for sensory evaluation. Five 50 mL conical bottles containing 20 mL raw soy sauce were prepared and randomly numbered for sensory scoring during the sensory evaluation. The sensory attributes of the soy sauce regarding appearance, aroma, taste, and overall acceptability were evaluated by 10 evaluators who scored each item and calculated the average value. Color, aroma, taste, and body were scored 25, with a total score of 100. The 60 points for low-salt solid-state fermented soy sauce sold in the market were used as the evaluation benchmark to clarify the sensory grade expressed by the corresponding score. 

### 2.8. Statistical Analysis

All experimental data were statistically evaluated using one-way ANOVAs and Duncan’s post hoc tests. Data are given as the means ± SD, and significance was defined as *p* < 0.05 using IBM SPSS Statistics 24.0 (SPSS Inc., Chicago, IL, USA). All statistical analyses were processed with OriginPro 2021 (OriginLab, Northampton, MA, USA) software and Design Expert software package 8.0.5.0 (State-Ease, Inc., Minneapolis, MN, USA). Principal component analysis and orthogonal partial least squares discriminant analysis were performed in Simca 14.1 (version 14.1, Umetrics AB, Umea, Vasterbotten, Sweden).

## 3. Results and Discussion 

### 3.1. Determination of the Optimal Koji Collection Time and the Types and Ratios of Raw Materials

The growth of *A. oryzae* 3.042 during the preparation of koji largely determines the quality of the fermented soy sauce [26]. Here, the activities of protease, glucoamylase, and cellulase in the culture at 36 h, 40 h, and 44 h were determined to find out the optimal koji collecting time and the appropriate type and proportion of the raw materials. 

**Figure 1:**
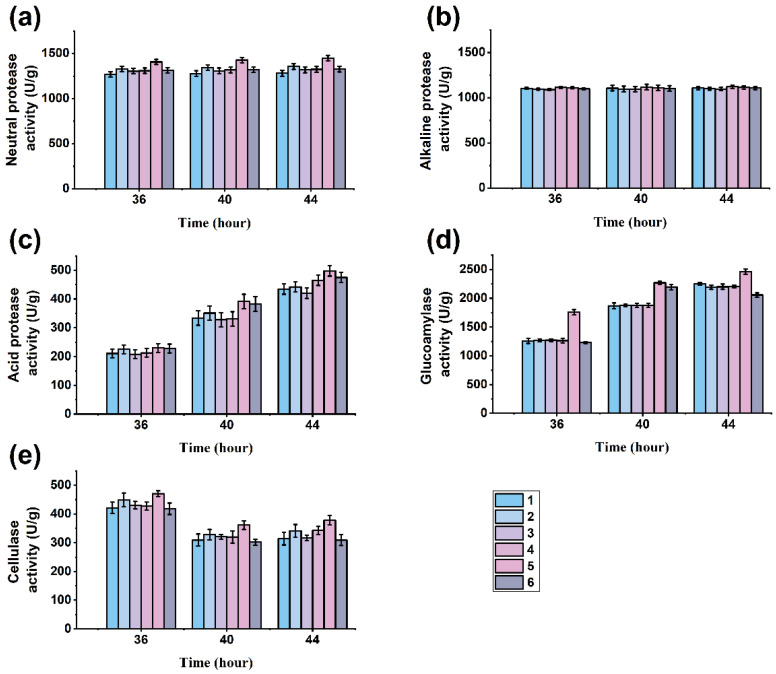
Enzyme activities of koji: (**a**) neutral protease; (**b**) alkaline protease; (**c**) acid protease; (**d**) glucoamylase; (**e**) cellulase. (Numbers 1–6 indicate different proportions of the raw materials: no. 1—soybean:fried wheat = 5:5; no. 2—soybean:fried wheat = 6:4; no. 3—soybean:fried wheat = 7:3; no. 4—soybean meal:fried wheat = 5:5; no. 5—soybean meal:fried wheat = 6:4; and no. 6—soybean meal:fried wheat = 7:3).

During the initial stage of soy sauce fermentation, *A. oryzae* secretes a significant number of enzymes, resulting in the highest utilization rate of the raw materials. Protease [27] is the most crucial enzyme system secreted in koji, as it fully hydrolyzes protein substances into nutrients. These nutrients contribute to the rich flavor and high quality of a soy sauce. Figure 1a–c show the results of different protease activities. During the period of 36–44 h, the protease activity of the koji increased gradually. The activities of the neutral protease, alkaline protease, and acid protease of the koji reached their peaks at 44 h, at which time the overall protease activity in group 5 was the highest, with neural, alkaline, and acid protease activities of 1422.53 U/g, 1122.53 U/g, and 500.61 U/g, respectively. The alkaline and neutral proteases were the most active in the initial system due to the generally high pH at the beginning of the fermentation. As the fermentation progressed, the pH of the system gradually decreased, leading to an increase in acid protease activity [21]. The raw soybean meal material was defatted of extruded soybeans, because after extrusion treatment, it is distributed in flakes, without the three-dimensional particles of soybeans [28]. Moderate deformation can increase the contact point between the raw material and protease to improve efficiency. And the moderate amount of the raw fried wheat material hydrolyzed by amylase produces glucose, which can provide energy for *A. oryzae*, so the ratio of the raw material in group 5 had a higher protease activity.

In the fermentation process of soy sauce, glucoamylase is crucial to the hydrolysis of the raw materials, which directly affects the utilization rate of the raw materials and the quality of soy sauce. Glucoamylase hydrolyzes starch from the nonreducing, nonterminal a-1.4 glucosidic bond to produce glucose and also slowly hydrolyzes the a-1.6 glucosidic bond to convert it into glucose. Glucoamylase hydrolyzes starchy raw materials into monosaccharides such as glucose, which provide carbon sources for microorganisms during soy sauce fermentation and participate in the Maillard reaction, thus affecting the flavor and taste of soy sauce [29]. As shown in Figure 1d, the glucoamylase activity of koji generally showed an upward trend during 36~44 h of koji culture. When cultured for 44 h, the glucoamylase activities of all groups reached the maximum. Moreover, the glucoamylase activity of group 5 was 2461.55 U/g, which is higher than that of the other experimental groups. Therefore, it was confirmed that the time of the koji collection was the 44th hour.

Cellulase is a compound enzyme that catalyzes the hydrolysis of the β-1,4 glucosidic bond within cellulose to cellobiose and glucose and improves the quality of fermented soy sauce by accumulating these substances. However, if *A. oryzae* overproduces cellulose, it can lead to excessive hydrolysis of cellobiose and glucose. In that case, it will combine with the protease and amylase decompositions of FAAs and disaccharides to produce harmful substances, reducing the content of salt-free solids in the finished soy sauce [18]. As shown in Figure 1e, the cellulase activity showed a declining trend during 36~44 h in the koji. The cellulase enzyme activity was lower at 44 h, so harvesting at 44 h was more appropriate. The cellulase enzyme activity of experimental group 5 was the highest at 44 h, reaching 387.22 U/g. Therefore, the cellulase enzyme activity of experimental group 5 was more suitable for soy sauce fermentation.

The final determination of the raw material types was soybean meal and fried wheat mixed at a ratio of 6:4, and the optimal koji collection time was 44 h, taking into account several enzymatic activities of the koji and the inexpensive characteristics of the raw soybean meal material.

### 3.2. Determination of Strains and the Additional Amount 

**Figure 2:**
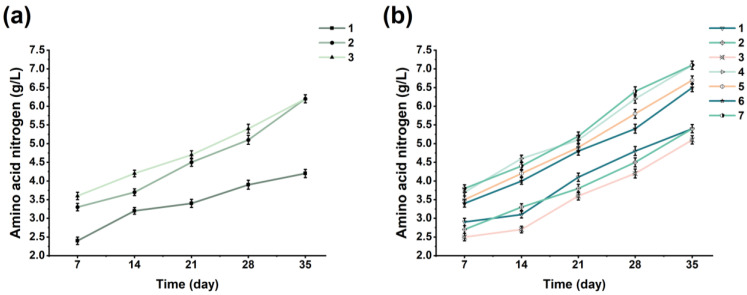
The variation in the amino acid nitrogen during fermentation with Lactobacillus and yeast: (**a**) addition of *T. halophilus*—no. 1: 0 CFU/g, no. 2: 1.0 × 10^6^ CFU/g, and no: 3: 3.0 × 10^6^ CFU/g; (**b**) addition of different kinds of yeast—no. 1: *Z. rouxii*, no. 2: *T. halophilus*, no. 3: *C. versatilis*, No. 4: *Z. rouxii* + *T. halophilus*, No. 5: *Z. rouxii* + *C. versatilis*, No. 6: *T. halophilus* + *C. versatili*, and No. 7: *Z. rouxii* + *T. halophilus* + *C. versatili*.

The effect of LAB on the whole sauce mash system is the conversion of sugars into lactic acid, thereby inhibiting the growth of other microorganisms and improving the shelf life of products. Adding LAB during soy sauce fermentation can change the pH of the soy sauce mash system to one more conducive to the growth of yeasts added later. After ultrasonic treatment of the sauce mash in the first three days, *T. halophilus* was added on the third day. It can be seen from Figure 2a that the addition of *T. halophilus* can improve the AAN content of soy sauce. After fermentation, the final AAN contents of the 1.0 × 10^6^ CFU/g *T. halophilus* fermentation group and the 3.0 × 10^6^ CFU/g *T. halophilus* fermentation group were 6.30 g/L and 6.40 g/L, respectively, with no significant difference. In addition, both fermentation groups reached pH values below 5.0 on the 7th day. Consequently, 1.0 × 10^6^ CFU/g *T. halophilus* was selected for economic reasons.

To improve the quality of the short-term fermentation of soy sauce, different yeasts and their combinations were added in this study. The main types of yeast were *Z. rouxii*, *T. halophilus*, and *C. versatilis*. It can be seen from Figure 2b that the addition of six different kinds of yeast in the early stage of soy sauce fermentation promoted the accumulation of amino acid nitrogen content. At the end of the fermentation, the AAN contents of the No. 7 soy sauce and No. 4 soy sauce were both 7.10 g/L, which is higher than that of the other groups. However, there was no significant difference among the combined yeasts addition groups, so *Z. rouxii* and *T. halophilus* were selected because of economic consideration. Among the single addition groups, the effect of *Z. rouxii* was better than that of *T. halophilus*, and both were better than that of *C. versatilis*. *Z. rouxii* and *T. halophilus* are the most frequently added yeasts in the HS soy sauce process. The main reason is that *Z. rouxii* and *T. halophilus* can make the raw materials more fully decompose, and *Z. rouxii* can be fermented by glucose, maltose, and fructose. *T. halophilus* was mainly used in the late stage of sauce mash fermentation, which can produce a large number of phenolic substances and other flavoring substances causing fermented soy sauce to possess a strong sauce flavor and a large number of flavor substances. The mixed addition of *Z. rouxii* and *T. halophilus* and the mixed addition of *Z. rouxii*, *T. halophilus,* and *C. versatilis* improved the accumulation of AAN contents in the LS soy sauce in a short amount of time. However, the effect of three kinds of yeast mixed with *Z. rouxii* and *T. halophilus* on the soy sauce mash was basically the same. Therefore, the mixed addition of *Z. rouxii* and *T. halophilus* in equal proportions was applied to the fermentation process.

### 3.3. Determination of New Fermentation Technology

**Figure 3:**
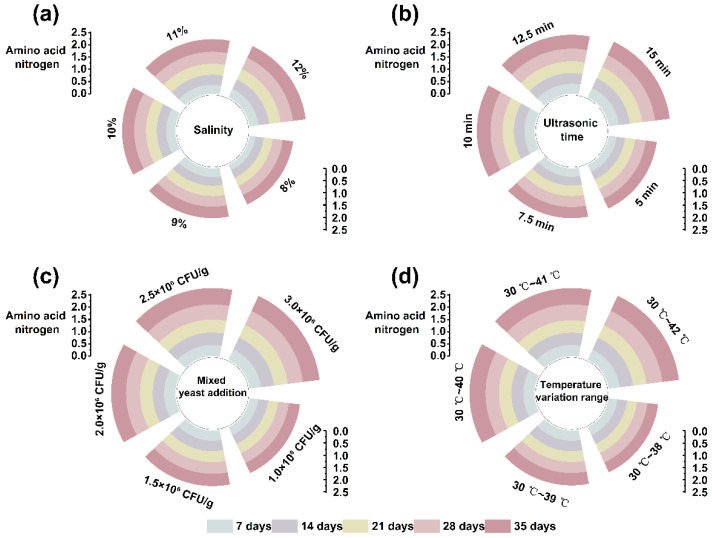
The variation in the amino acid nitrogen contents during fermentation with different (**a**) salinities; (**b**) ultrasonic times; (**c**) mixed yeast additions; (**d**) temperature variation ranges during fermentation.

In order to determine the amount of salinity, ultrasonic time, mixed yeast addition, and the temperature variation range, the AAN content was taken as the reference basis, and the results are shown in Figure 3. 

This study aimed to develop a reduced-salt soy sauce that will ensure a healthy diet for most people without losing the quality of the soy sauce. As can be seen from Figure 3a, the AAN contents of the five groups with different salt additions gradually increased during the fermentation of the soy sauces in the early fermentation stages and became stable later. However, the increase in the AAN content in the soy sauce when the salinity was 10% showed a significant difference, which indicates that the increase in the salinity increases the AAN content to some extent. Therefore, 9%, 10%, and 11% salinity levels were selected for the response surface analysis to determine the optimal salt addition level.

The application of ultrasound can significantly improve the accumulation of AAN at the prefermentation stage, but the length of the ultrasonic time also affects the fermentation; too long leads to excessive cracking of the raw materials, affecting the later fermentation. In Figure 3b, it can be seen that the AAN content was significantly different when the ultrasonic time was 10 min. Therefore, 7.5 min, 10 min, and 12.5 min of ultrasound were selected for the response surface analysis to determine the optimal ultrasonic time for the first three days of fermentation. 

The mixed yeast addition also affected the AAN content of the soy sauce during fermentation. It can be seen from Figure 3c that all five mixed yeast additions had a promoting effect on the AAN content of the soy sauce, increasing the most when the amount of *Z. rouxii* and *T. halophilus* added to the mixture was 2.0 × 10^6^ CFU/g, showing a significant difference. Therefore, the mixed yeast addition amounts of 1.5 × 10^6^ CFU/g, 2.0 × 10^6^ CFU/g, and 2.5 × 10^6^ CFU/g were selected for the response surface analysis to determine the optimal mixed yeast addition amounts.

Fermentation temperature plays a crucial role in the fermentation of soy sauce. LS soy sauce is mainly fermented at high temperatures for a short time, resulting in poor flavor and a dark color; HS soy sauce is mainly fermented continuously at low temperature, which provides a rich in flavor but has a long fermentation time, resulting in high selling prices. This SR soy sauce was fermented at 42 °C for 7 days, cooled to 30 °C, and then warmed, so the temperature change in the later warmed-up fermentation is essential. Figure 3d shows that the AAN contents of the five different temperatures for the fermentation of soy sauce differed significantly, starting with an initial temperature of 42 °C for 7 days, followed by cooling to 30 °C, and then rising 1 °C every two days to 40 °C during continuous fermentation. Therefore, the late-fermentation temperature variations of 30 °C~39 °C, 30 °C~40 °C, and 30 °C~41 °C were selected for the response surface analysis to determine the best late fermentation variation in temperature.

The response surface method was used to optimize the experimental results, as shown in Tables S1 and S2. According to Table S2, the *p*-value of the regression model was less than 0.01, which is a highly significant level, indicating that the model was significantly regressed, and the regression equation well represented the relationship between the amino acid nitrogen content and the four factors. The multiple correlation coefficient R^2^ was 0.8917, indicating a good fit between the measured and predicted values. The *p*-value of the misfit term in the table (0.5630) was greater than 0.05, indicating that it was not significant. This demonstrates that the AAN was influenced by these four fermentation conditions rather than the noise in the domain. The regression model’s significance test and ANOVA results show that the regression model obtained by adopting the response surface method in the experimental design was valid and suitable for the optimization test of the fermentation conditions of the SR soy sauce. The partial regression coefficients of A^2^, B^2^, C^2^, and D^2^ in the quadratic terms reached the highly significant level. A quadratic response surface regression analysis was performed on the experimental data using Design Expert 8.0 software, and the simulated equations for the AAN content of the soy sauce with each dependent variable were obtained as follows:

(2) $$Y=0.85+0.000A+0.018B+0.040C+0.037D-0.047AB+0.013AC+0.010AD\;+0.017BC-0.020BD-0.020CD-0.14A^{2}-0.12B^{2}-0.14C^{2}-0.15D^{2}$$

where *Y* is the AAN, *A* is the salinity, *B* is the ultrasonic time, *C* is the mixed yeast addition, and *D* is the temperature variation range.

The optimal solution of the model was obtained with a salinity of 10.15%, ultrasonic time of 10.2 min, mixed yeast addition amount of 2.40 × 10^6^ CFU/g, and temperature variation range of 30~41.2 °C. To test the reliability of the results obtained by the response surface methodology, an actual fermentation process was carried out under the following conditions: salinity of 10%, ultrasonic time of 10 min, mixed yeast addition of 2.50 × 10^6^ CFU/g, and variable temperature value of 30~41 °C. The average AAN contents of the SR soy sauce products produced by fermentation under these conditions was 8.40 g/L for three replicate experiments, consistent with the theoretical prediction. Therefore, the optimal fermentation process conditions obtained on the basis of the response surface method are realistic, reliable, and have practical production value.

Taken together, a new fermentation process was established. Briefly, seed koji was inoculated in a 6:4 mixture of steamed soybean meal and fried wheat and cultured for 44 h. The koji was then collected, mixed with 10% saline for soy sauce fermentation, and sonicated for 10 min from day 1 to day 3. Lactic acid bacteria (1.00 × 10^6^ CFU/g) and the mixture of *Z. rouxii* and *C. versatilis* (1.25 × 10^6^ CFU/g each) were separately added on the third and seventh days. The initial fermentation temperature was 42 °C for 7 days, then cooled down to 30 °C and increased by 0.5 °C daily to 41 °C until the end of the fermentation. The entire soy sauce mash fermentation cycle of the new process was 35 days. A comprehensive comparative study was conducted on the qualities of the soy sauce samples fermented by the new fermentation process and traditional LS and HS fermentations (20% and 12% saline for HS and LS, respectively). The indicators for comparison included the AAN, TN, RS, salt content, color indexes, organic acids, FAAs, and flavor substances. 

### 3.4. Comparison of the Physical and Chemical Indexes of Different Kinds of Soy Sauces 

The content of AAN is an important index that can measure the grade of a soy sauce [30]. During the fermentation process of soy sauce, large molecules of protein are hydrolyzed into peptides, which are further hydrolyzed into small molecules of FAAs, as the main taste-presenting substances in soy sauce directly determine its flavor and taste [31]. The changes in AAN contents during fermentation are shown in Table 1. The AAN content of the SR soy sauce was 8.20 g/L, and that of the HS soy sauce was 9.10 g/L. The AAN content of the LS soy sauce (LS) was significantly lower than that of the above two at only 4.80 g/L. This was due to the short fermentation cycle of the LS resulting in AAN accumulation, while the SR soy sauce significantly improved the drawbacks of traditional LS fermentation by adding LAB and ultrasound in the early stage of fermentation. These results show that the AAN level of the SR soy sauce was significantly higher than that of the LS soy sauce and basically the same as that of the HS soy sauce.

TN is the nitrogen content of all soluble nitrogen compounds, which reflects the quality of the soy sauce and protein utilization. The TN content of soy sauce from three fermentation groups are shown in Table 1. Consistent with the results for the AAN, the TN content of the soy sauce in each group was 12.5 g/L for the LS soy sauce, 14.9 g/L for the SR soy sauce, and 15.2 g/L for the HS soy sauce. The TN content of the SR soy sauce was significantly higher than that of the LS soy sauce, mainly due to the high initial fermentation temperature and the use of ultrasound to accelerate the cracking of the raw materials, which resulted in the rapid accumulation of the TN content.

The content of RS determines the sweet and salty taste of soy sauce and affects the taste of finished soy sauce [32]. In soy sauce fermentation, amylase and glucoamylase hydrolyze the starch in raw materials into RS. The results are shown in Table 1. The RS content and enzyme activity in the SR soy sauce fermentation were higher in comparison to the three different soy sauce fermentation methods. Generally, the amount of RS increases at the beginning of fermentation, as *A. oryzae* gradually breaks down the raw material. However, as the fermentation progressed, the viability of *A. oryzae* gradually decreased, and yeast and LAB started to use the RS in the fermentation system, resulting in a significant decrease in the RS content in the later stages [33], which is the reason why the RS contents of the HS and SR groups were lower than that of the LS group.

### 3.5. Salt Content of SR Soy Sauce in Comparison to HS Soy Sauce and LS Soy Sauce

The salt content after fermentation is shown in Table 1. The final salt content of the SR soy sauce was 91.0 g/L, much lower than that of the HS soy sauce. The salt content of this soy sauce was equivalent to that of commercial salt-reduced soy sauce, achieving a salt reduction of 35% and meeting the standard for salt-reduced soy sauce. Hu et al. [34] studied fermented soy sauce with different salt concentrations. The results indicated that as the salt content decreased, without any other process improvements, the amount of AAN also decreased, with the lowest AAN content being 0.31 g/mL. In this study, the AAN level of the SR soy sauce did not decrease with the reduction in salt content. Its AAN content was higher than that of the LS soy sauce and close to the HS soy sauce index, indicating that the SR soy sauce did not reduce the salt content at the expense of quality.

Reducing the amount of salt in soy sauce products has been reported to boost the content of isoflavones, which are the main bioactive substances in soy sauce products [35]. Isoflavones are phytoestrogens and good antioxidant components, and some studies have shown anti-inflammatory and antibacterial properties. Studies have found that they may also play a role in the prevention of some chronic diseases, including heart disease and cognitive impairment. This is another indication that SR soy sauce plays a positive role in human health [36,37].

### 3.6. Distribution of the Different Organic Acids Contents among the Three Soy Sauce Samples

**Figure 4:**
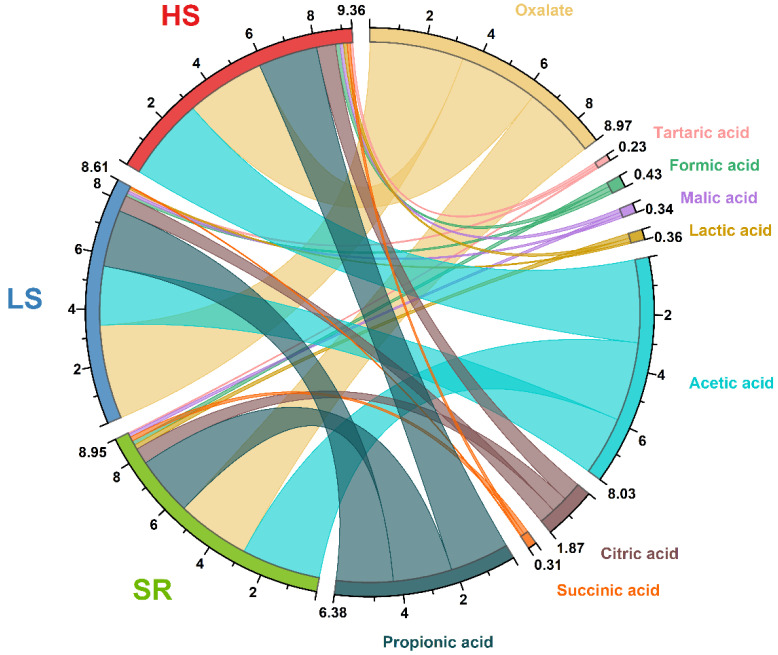
Comparison of the contents of nine organic acids among three soy sauces. The red, blue, and green rings on the left depict several types of soy sauce. The different colored rings and cords on the right side indicate different organic acids. The widths of the cords represent the contents of organic acids (mg/mL).

Organic acids can enrich the flavor of soy sauce [38]. The organic acids in soy sauce are mainly added with lactic acid bacteria to make part of the sugar become lactic acid. The contents of organic acids determine the sour taste of soy sauce. In addition, the production of ester during fermentation is also closely related to organic acids, and it directly determines the quality of soy sauce. As shown in Figure 4 and Table S3, some organic acids, including propionic acid and acetic acid, were higher in the HS soy sauce, which was not much different from the SR soy sauce. Oxalic acid harms the human body, so its content in the finished soy sauce is required to be lower. The oxalic acid content in the soy sauce samples were SR soy sauce < HS soy sauce < LS soy sauce, and the oxalic acid accounted for the lowest proportion of total organic acids among the three samples. Acetic acid is a common organic acid in soy sauce, mainly in the form of lipids. The acetic acid content of the SR soy sauce was higher than that of the LS soy sauce. An appropriate amount of acetic acid is beneficial to human health and can regulate the taste of soy sauce, making soy sauce taste better. Propionic acid has an antisepsis effect on soy sauce. From the figure, the propionic acid content in the SR soy sauce was higher than in the LS soy sauce. For soy sauce with a relatively low salt content, propionic acid can compensate for contamination problems caused by insufficient salt content. As shown in Figure 4, the contents of organic acids in the new SR soy sauce were comparable to those in the HS soy sauce and significantly higher than those in the LS soy sauce. 

### 3.7. Comparison of the Free Amino Acid Compositions of the Different Kinds of Soy Sauce

**Figure 5:**
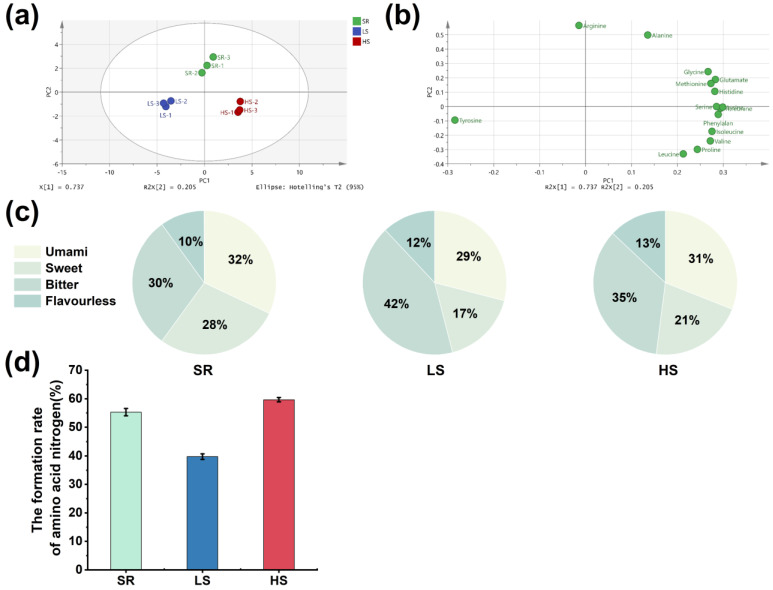
(**a**) PCA score plot and (**b**) PCA load diagram for 16 FAAs; (**c**) distributions of free amino acids regarding taste among the three kinds of soy sauce; (**d**) formation rates of AAN among the three kinds of soy sauce.

FAAs are gradually released from raw materials through enzyme catalysis, which is closely related to the taste of soy sauce [39,40]. Principal component analysis (PCA) was performed according to the results of the FAAs determination. The PCA showed that the FAAs in the three kinds of soy sauce were quite different. According to Figure 5a, there are apparent zoning features. The HS soy sauce was located in PC1’s positive area, the SR soy sauce was in PC2’s positive area, and the LS soy sauce was significantly different from the above two kinds of soy sauce in PC1’s and PC2’s negative areas. As shown in Figure 5b, the PCA load diagram suggests that 16 FAAs are mainly distributed in the first and fourth quadrants, mainly concentrated in the first quadrant. From PC1, 14 FAAs were positively loaded, and the other two FAAs, arginine and tyrosine, were negatively loaded. The results show that the 14 FAAs in the positive and negative charges were the main representatives of the HS soy sauce. From the perspective of PC2, seven FAAs, including alanine, arginine, and glutamate, were the main FAAs in the SR soy sauce. Moreover, tyrosine was the main component of FAAs in the LS soy sauce.

According to the taste of the FAAs (umami, sweet, bitter, and flavorless), the three kinds of soy sauce were analyzed by the relative contents of the flavor substances. On the basis of the analysis of the relative contents of the flavor substances in Figure 5c, the SR soy sauce was higher than the other two groups in terms of umami, which shows that this SR soy sauce was better in terms of umami. Regarding sweetness, the SR soy sauce was on the sweet side, while the LS and HS soy sauces were slightly less sweet. In terms of bitterness, the LS soy sauce had high FAA contents, which might be related to the continuous high-temperature fermentation of the LS technology. The other two groups had lower bitter FAA contents. In terms of unflavored FAAs, the three groups of soy sauce were basically the same. 

The formation rate of AAN can be a good indicator of the degree of protein decomposition in raw materials and the economics of soy sauce [41]. The determined AAN production rates of the three kinds of soy sauce are shown in Figure 5d. As can be seen in Figure 5d, by comparing the three soy sauces, the AAN production rate of the SR soy sauce was 53%, which is significantly higher than that of the LS soy sauce, and it was basically the same compared with that of the HS soy sauce. 

Overall, the SR soy sauce is similar to the HS soy sauce in terms of the amino acid distribution, with a reduced salt content, substantially lower production cost, and practical application value.

### 3.8. Comparison of the Flavor Differences among the Different Kinds of Soy Sauce

The flavor compounds in the three fermentation groups were detected, and the main types are shown in Table 2 and Table S4. The main flavor substances of soy sauce are derived from protein, carbohydrates, fat, etc., and catalyzed by *A. oryzae*, salt-tolerant yeast, and salt-tolerant LAB. Alcohols, acids, esters, and aldehydes are the main flavor substances in soy sauce, of which alcohols are the most abundant. The high content of alcohol is due to the autolysis of the raw materials and the decomposition of sugars by yeast. Among the three kinds of soy sauce, the contents of flavor compounds in the SR soy sauce were basically the same as those in the HS soy sauce. Specifically, the lipid substances in the SR soy sauce were higher than those of the other two kinds of soy sauce, and the ester substances can promote the formation of a variety of soy sauce flavors and provide the fermented soy sauce with a solid, special sauce flavor. The acids were mainly organic acids; a few organic acids make a soy sauce’s flavor richer, but too many lead to a sour soy sauce, affecting the taste. The high contents of acids in soy sauce produced by the LS fermentation may be due to the rapid decomposition of the raw materials and the accumulation of acids. It can be seen in Table 2 and Table S4 that the contents of alcohols, esters, aldehydes, ketones, acids, furans, pyrroles, and other substances in the SR soy sauce were comparable to those in the HS fermentation process. These results indicate that the flavor of the SR soy sauce was more diversified and greatly improved with the optimized fermentation process based on the premise of reducing the salt content and shortening the fermentation cycle.

### 3.9. Sensory Evaluation of the Different Kinds of Soy Sauce

**Figure 6:**
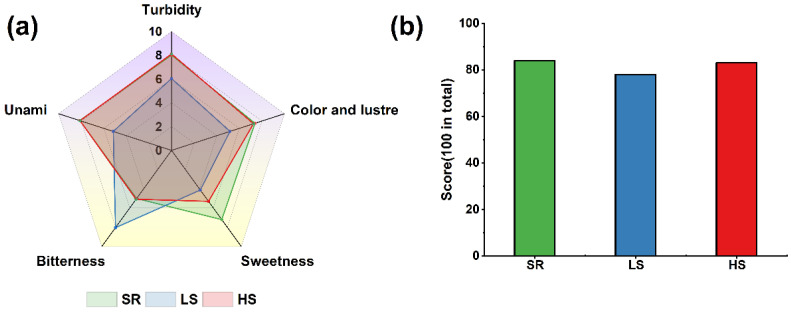
Results of the (**a**) sensory evaluation and (**b**) scores of the three kinds of soy sauce.

Sensory evaluation personnel evaluated three kinds of soy sauce from four aspects: color, posture, aroma, and taste. The evaluation results are shown in Figure 6a. The bitter taste of the LS soy sauce was heavier, but its color tended to be reddish brown. The colors and tastes of the SR and LS soy sauces were slightly different from that of the HS soy sauce, indicating that adding LAB and flavoring yeast to the fermentation process could improve the quality and smell of soy sauce in the short-term fermentation process.

According to Figure 6b, the SR and HS soy sauces’ scores were higher, and the LS soy sauce had the lowest. It can be seen that the quality of the HS soy sauce was better than that of the LS soy sauce. However, by optimizing the fermentation conditions and changing the fermentation temperature, the SR process achieved a good fermentation effect comparable to the overall index of the LS soy sauce. 

## 4. Conclusions

The above results demonstrate that the best fermentation method for the SR soy sauce was determined by optimizing different parts of the soy sauce fermentation process. The salt content of the SR soy sauce was 9.10 g/100 mL, which was significantly lower than that of the HS soy sauce. The AAN content of the SR soy sauce met the particular soy sauce standard, and the value was 0.84 g/100 mL, which is in line with the particular soy sauce standard in China. The flavor substances of the SR soy sauce did not differ significantly from those of the HS soy sauce based on the shortening of the fermentation cycle, and the flavor substances reached more than 80 kinds. In terms of the FAAs and organic acids contents, the SR soy sauce basically reached the level of the HS soy sauce. The SR soy sauce in this study had the same level of quality as the fermented in the HS soy sauce, with a reduced fermentation time and savings in terms of production costs, bridging the drawbacks of the reduced-salt soy sauce fermentation process. 

## Figures and Tables

**Table 1:** Comparison of the indexes among the three soy sauces.

	SR	LS	HS
Amino acid nitrogen (g/L)	8.20 ± 0.41 ^b^	4.80 ± 0.24 ^c^	9.10 ± 0.46 ^a^
Total nitrogen (g/L)	14.80 ± 0.74 ^b^	12.10 ± 0.61 ^c^	15.30 ± 0.77 ^a^
Reducing sugar (g/L)	22.12 ± 1.11 ^b^	22.90 ± 1.15 ^a^	14.85 ± 0.74 ^c^
Salt content (g/L)	91.00 ± 4.55 ^c^	124.00 ± 6.20 ^b^	223.00 ± 11.15 ^a^

Data are expressed as the means ± standard deviation. Different letters within the same row indicate significant differences (*p* < 0.05).

**Table 2:** The kinds and relative contents of flavor components by GC-MS.

Component	SR		LS		HS	
	Kinds	Relative Content (%)	Kinds	Relative Content (%)	Kinds	Relative Content (%)
Alcohols	8	52.47	11	38.85	12	53.06
Acids	5	40.59	6	49.26	5	38.52
Aldehydes	5	13.73	8	7.80	8	14.91
Esters	11	22.14	6	12.56	6	21.43
Ketones	2	8.50	2	6.72	2	11.24
Phenols	1	7.16	1	4.93	1	6.82
Furans	2	2.11	1	0.82	1	0.86
Pyrazines	2	3.61	1	1.54	2	2.25
Pyrroles	1	1.22	0	0	1	0.74

## Data Availability

The original contributions presented in the study are included in the article/Supplementary Material, further inquiries can be directed to the corresponding author.

## Supplementary Materials

The following supporting information can be downloaded at: https://www.mdpi.com/article/10.3390/foods13060971/s1, Table S1: Response to interview test plan and results; Table S2: The significance test and variance analysis of the regression model; Table S3: Determination of organic acid content in three soy sauces; Table S4: Substances included in the three groups of GC-MS results and their relative content.

## Abbreviations

AAN	Amino acid nitrogen
FAAs	Free amino acids
HS	High-salt liquid-state fermentation
LAB	Lactic acid bacteria
LS	Low-salt solid-state fermentation
RS	Reducing sugars
SR	New salt-reduced fermentation
TN	Total nitrogen
