# Supplementary material for: A Method of Reducing Salt Content in Fermented Soy Sauce Improves Its Flavor and Quality
Source: Foods. 2024 Mar 21;13(6):971. doi: 10.3390/foods13060971 (PMC10969990; doi:10.3390/foods13060971)
Supplement: Supplementary file 1 [file foods-13-00971-s001.zip › foods-2908080-supplementary.pdf]

Table S.1 Response to interview test plan and results

Number	A：Salinity (%)	B：Ultrasonic time (min）	C：Yeast inoculum quantity (CFU/mL)	D：Temperature range (℃)	Amino acid nitrogen (g/100 mL)
1	-1	-1	0	0	0.51
2	1	-1	0	0	0.63
3	-1	1	0	0	0.64
4	1	1	0	0	0.57
5	0	0	-1	-1	0.49
6	0	0	1	-1	0.67
7	0	0	-1	1	0.56
8	0	0	1	1	0.68
9	-1	0	0	-1	0.55
10	1	0	0	-1	0.48
11	-1	0	0	1	0.54
12	1	0	0	1	0.52
13	0	-1	-1	0	0.52
14	0	1	-1	0	0.51
15	0	-1	1	0	0.56
16	0	1	1	0	0.62
17	-1	0	-1	0	0.56
18	1	0	-1	0	0.51
19	-1	0	1	0	0.62
20	1	0	1	0	0.67
21	0	-1	0	-1	0.52
22	0	1	0	-1	0.61
23	0	-1	0	1	0.66
24	0	1	0	1	0.67
25	0	0	0	0	0.85
26	0	0	0	0	0.78
27	0	0	0	0	0.81
28	0	0	0	0	0.93
29	0	0	0	0	0.86

Table S.2 The significance test and variance analysis of the regression model

Source	Sum of squares	Degrees of freedom	Mean square	F-value	*P*-value	Source
Model	0.36	14	0.026	8.23	0.0002	*
A Salinity	0	1	0	0	1	
B Ultrasonic time	4.03×10^{3}	1	4.03×10^{3}	1.28	0.2774	
C Mixed yeast addition	0.019	1	0.019	6.08	0.0272	
D Temperature variation range	0.016	1	0.016	5.11	0.0403	*
AB	9.03×10^{3}	1	9.03×10^{3}	2.86	0.113	
AC	6.25×10^{4}	1	6.25×10^{4}	0.2	0.6632	
AD	4.00×10^{4}	1	4.00×10^{4}	0.13	0.7272	
BC	1.23×10^{3}	1	1.23×10^{3}	0.39	0.5434	
BD	1.60×10^{3}	1	1.60×10^{3}	0.51	0.4882	
CD	1.60×10^{3}	1	1.60×10^{3}	0.51	0.4882	
A^2	0.13	1	0.13	41.28	< 0.0001	**
B^2	0.092	1	0.092	29.21	< 0.0001	**
C^2	0.12	1	0.12	38.42	< 0.0001	**
D^2	0.14	1	0.14	44.24	< 0.0001	**
Residual	0.044	14	3.16×10^{3}			
Lack of Fit	0.031	10	3.13×10^{3}	0.97	0.5630	
Pure Error	0.013	4	3.23×10^{3}			
Cor Total	0.41	28				
R^2=0.8917，R^2_{Adj}=0.7834						

Note: Blank, no significant difference; * Significant difference P<0.05；** the difference was highly significant P<0.01.

Table S.3 Determination of organic acid content in three soy sauces

Organic acid (mg/mL)	SR	LS	HS
Oxalate	2.67±0.13^{b}	3.43±0.17^{a}	2.87±0.14^{b}
Tartaric acid	0.05±0.0025^{c}	0.08±0.0048^{a}	0.10±0.0054^{a}
Formic acid	0.16±0.013^{a}	0.11±0.0051^{b}	0.16±0.0080^{a}
Malic acid	0.12±0.0060^{a}	0.09±0.0045^{b}	0.13±0.0065^{a}
Lactic acid	0.17±0.0085^{a}	0.06±0.0035^{c}	0.13±0.0065^{b}
Acetic acid	2.88±0.14^{b}	2.14±0.11^{c}	3.01±0.15^{a}
Citric acid	0.59±0.030^{b}	0.59±0.033^{b}	0.69±0.042^{a}
Fumaric acid	ND	ND	ND
Succinic acid	0.16±0.0081^{a}	0.04±0.0024^{c}	0.11±0.0055^{b}
Propionic acid	2.15±0.11^{a}	2.07±0.10^{b}	2.16±0.16^{a}
Total	8.95±0.69^{a}	8.61±0.38^{b}	9.36±0.43^{a}

*: "ND" means not detected. Different letters within the same row indicate significant differences ($p < 0.05$).

Table S.4 Substances included in the three groups of GC-MS results and their relative content.

Component	SR	LS	HS
ethanol	12.62	9.28	10.53
2-methyl-1-butanol	6.5	3.64	5.45
3-methyl-1-butanol	9.34	6.51	8.83
1-pentanol	ND*	ND	2.63
2,3-butanediol	12.83	6.49	8.22
furfuryl alcohol	4.57	2.39	3.48
hexyl alcohol	ND	ND	1.69
5-methyl-2-furanmethanol	1.69	1.88	1.27
1-heptanol	ND	ND	1.62
3-methylthiopropanol	2.65	2.33	2.99
1-octen-3-ol	ND	ND	2.88
5-methyl-5-nonanol	ND	2.52	ND
3,5-dimethyl-hexane-1,3,4-triol	ND	3.81	0.82
1-octanol	ND	1.66	ND
phenylethyl alcohol	2.27	1.04	2.65
**Relative content of alcohols**	52.47	38.85	53.06
**Number of alcohols**	8	11	12
acetic acid	18.66	15.37	16.59
isobutyric acid	3.51	4.67	4.38
isovaleric acid	6.32	8.86	8.21
2-methylbutyric acid	ND	6.23	ND
4-methylvaleric acid	ND	4.89	ND
heptanoic acid	ND	ND	1.99
octanoic acid	3.42	ND	ND
palmitic acid	8.68	9.24	7.35
**Relative content of acids**	40.59	49.26	38.52
**Number of acids**	5	6	5
isobutyraldehyde	1.52	0.68	1.74
isobutyralbegyde	4.7	2.22	4.26
tiglic aldehyde	ND	ND	0.23
furfural	1.19	0.34	1.66
heptaldehyde	ND	ND	0.29
methional	ND	ND	0.14
benzaldehyde	2.87	1.52	2.91
benzeneacetaldehyde	3.45	1.16	3.68
1-nonanal	ND	1.01	ND
decanal	ND	0.37	ND
2,4-dimethylbenzaldehyde	ND	0.5	ND
**Relative content of aldehydes**	13.73	7.8	14.91

**Number of aldehydes**	5	8	8
methyl acetate	3.81	ND	3.56
isobutyl acetate	ND	2.46	ND
isoamyl acetate	1.93	1.95	2.12
2-methylbutyl acetate	0.86	ND	ND
4-hydroxybutyric acid	1.05	1.14	ND
methyl hexanoate	3.75	ND	3.64
vinyl hexanoate	0.69	ND	ND
methyl benzoate	0.94	ND	ND
phenethyl acetate	3.64	2.2	4.86
methyl hexadecanoate	ND	2.18	ND
ethyl palmitate	2.09	ND	3.68
methyl linoleate	3.38	2.63	3.57
**Relative content of esters**	22.14	12.56	21.43
**Number of esters**	11	6	6
2,3-pentanedione	3.89	ND	5.38
3-octanone	ND	ND	5.86
2,3-octanedione	4.61	3.98	ND
2,5-dimethyl-3-hexanone	ND	2.74	ND
**Relative content of ketones**	8.50	6.72	11.24
**Number of ketones**	2	2	2
guaiacol	7.16	ND	6.82
4-hydroxy-3-methoxystyrene	ND	4.93	ND
**Relative phenols content**	7.16	4.93	6.82
**Number of phenols**	1	1	1
2,5-dimethylfuran	1.06	0.82	0.86
2-pentylfuran	1.05	ND	ND
**Relative content of furans**	2.11	0.82	0.86
**Number of furans**	2	1	1
2,6-dimethylpyrazine	1.85	1.54	1.62
2,3,5-trimethylpyrazine	1.76	ND	0.63
**Relative content of pyrazines**	3.61	1.54	2.25
**Number of pyrazines**	2	1	2
2-acetyl pyrrole	1.22	ND	0.74
**Relative content of pyrroles**	1.22	ND	0.74
**Number of pyrroles**	1	0	1

*: "ND" means not detected.
